# Maternal organokines throughout pregnancy as predictors of neonatal anthropometric characteristics and adiposity

**DOI:** 10.3389/fendo.2024.1423950

**Published:** 2024-12-04

**Authors:** Jorge Valencia-Ortega, Victoria Galicia-Hernández, Andrea Castillo-Santos, Miranda Molerés-Orduña, Carla Arceo-Cerna, Otilia Perichart-Perera, Ameyalli M. Rodríguez-Cano, Carolina Rodríguez-Hernández, Guadalupe Estrada-Gutierrez, Ignacio Camacho-Arroyo, Juan Mario Solis-Paredes

**Affiliations:** ^1^ Unidad de Investigación en Reproducción Humana, Instituto Nacional de Perinatología-Facultad de Química, Universidad Nacional Autónoma de México, Mexico City, Mexico; ^2^ Department of Reproductive and Perinatal Health Research, Instituto Nacional de Perinatología Isidro Espinosa de los Reyes, Mexico City, Mexico; ^3^ Sección de Estudios de Posgrado, Escuela Superior de Medicina, Instituto Politécnico Nacional, Mexico City, Mexico; ^4^ Nutrition and Bioprogramming Coordination, Instituto Nacional de Perinatología Isidro Espinosa de los Reyes, Mexico City, Mexico; ^5^ Department of Immunobiochemistry, Instituto Nacional de Perinatología Isidro Espinosa de los Reyes, Mexico City, Mexico

**Keywords:** gestational weight gain, maternal obesity, birth weight, neonatal adiposity, organokines

## Abstract

**Aims:**

To evaluate the relation between maternal concentrations of progranulin (PGRN), adipocyte fatty acid-binding protein (AFABP), brain-derived neurotrophic factor (BDNF), and fibroblast growth factor 21 (FGF21) throughout pregnancy with neonatal weight and length at birth and at one month of age, as well as with the percentage of fat mass at one month of age. Besides, we evaluated the association between maternal organokine concentrations with pregestational nutritional status and gestational weight gain (GWG).

**Methods:**

Longitudinal study of 100 healthy pregnant women and their neonates. Conventional biochemical tests were performed and maternal organokine concentrations were measured by ELISA. Neonatal percent fat mass was determined using the PEA POD system, and weight and length were measured using a soft tape measure and a baby scale. Multiple linear regression models were made to predict neonatal anthropometric measurements and adiposity.

**Results:**

In all women, PGRN concentrations significantly increased as pregnancy progressed, while AFABP concentrations increased until the third trimester and the highest BDNF concentrations were observed in the second trimester of pregnancy. In contrast, FGF21 concentrations did not change during pregnancy. Only maternal obesity was associated with some differences in AFABP and FGF21 concentrations. Gestational age at birth, maternal age and third-trimester PGRN concentrations predicted weight (gestational age at birth: β=0.11; maternal age: β=-0.033; PGRN: β=0.003, *p*<0.001) and, together with first-trimester BDNF concentrations, length (gestational age at birth: β=0.76; maternal age: β=-0.21; PGRN: β=0.24; BDNF: β=0.06, *p*<0.001) at birth. Maternal age and third-trimester BDNF concentrations predicted one-month-old neonate length (maternal age: β=-1.03; BDNF: β=0.45, *p*<0.001). Pregestational body mass index (pBMI), GWG, second-trimester FGF21 concentrations, and third-trimester AFABP concentrations predicted neonatal fat mass percentage (pBMI: β=-0.58; GWG: β=-0.32; FGF21: β=-0.004; AFABP: β=-1.27, *p*<0.001) at one month of age.

**Conclusion:**

Maternal PGRN, AFABP, and BDNF concentrations, but not FGF21, vary throughout pregnancy. These organokines and maternal characteristics can be useful in the prediction of neonatal weight, length, and percentage fat mass.

## 1 Introduction

The developmental origins of health and disease (DOHaD) hypothesis suggests that early life conditions of an individual (periconceptional, fetal, and early childhood stages) are linked to the long-term development of metabolic disorders (1). Birth weight is associated with the incidence of lifestyle-related disorders in adulthood, such as cardiovascular disease and diabetes (2). Maternal pregestational nutritional status and gestational weight gain (GWG) strongly influence birth weight. Maternal obesity increases the risk of macrosomia and low birth weight (3), while excessive GWG is associated with increased birth weight, and insufficient GWG is associated with low birth weight (4, 5). Besides, there is a positive correlation between birth weight and the percentage of fat mass in neonates (6). This percentage is also associated with adiposity and body mass index later in life (7).

Emerging evidence suggests that dysregulation in the crosstalk between organs such as the liver, muscle, adipose tissue, and placenta, through the production of protein messengers called organokines (including myokines, adipokines, and hepatokines), may participate in the relationship between maternal weight and neonatal anthropometric outcomes (8–11).

Leptin and adiponectin are the most studied organokines to date, while dozens of other organokines with the potential to modulate maternal metabolism remain unstudied throughout pregnancy, and their association with neonatal anthropometric outcomes has not been evaluated (12–14). Progranulin (PGRN) is a protein secreted by various cell types, including fibroblasts, adipocytes, and neurons (15). It has been implicated in several physiological and pathological processes, including glucose and lipid metabolism, inflammation, and insulin-resistance (16). Individuals with type 2 diabetes or obesity have increased plasma concentrations of PGRN (17). Adipocyte fatty acid-binding protein (AFABP) belongs to the fatty acid-binding protein family and is primarily expressed in adipose tissue. It plays a crucial role in systemic insulin sensitivity, glucose, and lipid metabolism, and its concentrations are linked to obesity, metabolic syndrome, and vascular disease (18, 19). Brain-derived neurotrophic factor (BDNF) is a protein member of the neurotrophin family produced mainly in the nervous system, but also in peripheral tissues such as skeletal muscle, liver and adipose tissue (20, 21). It regulates glucose metabolism in peripheral tissues, playing a role in energy homeostasis (22). Fibroblast growth factor 21 (FGF21) is a protein member of the fibroblast growth factor family produced by various tissues, including the liver, adipocytes, pancreas, and placenta (23, 24). In animals, FGF-21 plays a role in glucose and lipid metabolism (25). Increased circulating concentrations of this protein have been associated with obesity, insulin resistance, and type 2 diabetes (26, 27).

We hypothesized that during pregnancy, PGRN, AFABP, BDNF, and FGF21 regulate maternal glucose and lipid metabolism, which may ultimately influence nutrient availability to the developing fetus. This may result in a relation between the circulating concentrations of these organokines and neonatal anthropometry and adiposity. Additionally, it is possible that the concentrations of these organokines are associated with pregestational nutritional status and GWG.

To further investigate the potential role of these organokines in the relationship between maternal weight and neonatal anthropometric outcomes, the aim of the present study was to evaluate the relation between maternal concentrations of PGRN, AFABP, FGF21, and BDNF throughout pregnancy with neonatal weight and length at birth and at one month of age, as well as with the percentage of fat mass at one month of age. Besides, we evaluated the association between maternal organokine concentrations with pregestational maternal weight and GWG.

## 2 Material and methods

This study derives from the OBESO (Origen Bioquímico y Epigenético del Sobrepeso y la Obesidad) perinatal cohort conducted at the Instituto Nacional de Perinatología in Mexico City, Mexico. The research protocol was approved by the Institutional Research and Ethics Review Board (3300-11402-01-575-17 and 2019-1-20). The study was conducted in accordance with the Declaration of Helsinki ethical principles for human research. Participation was voluntary, and all participants signed an informed consent form.

### 2.1 Participants

Women were enrolled during the first trimester (11.0–13.6 weeks determined by ultrasound) of pregnancy with follow up at the second (18-24.6 weeks) and third (28-34.6) trimesters. Inclusion criteria were as follows: healthy adult pregnant women (≥18 years old), pregestational body mass index (pBMI) ≥18.5 kg/m^2^, single pregnancy, neonate at term, and exclusively breastfed over the first month of life. Women with pregestational comorbidities such as hypertension, uncontrolled thyroid disorders, diabetes mellitus, autoimmune diseases, kidney and liver diseases, and human papillomavirus and human immunodeficiency virus infection were not included. Development of pregnancy-related complications (i.e., gestational diabetes mellitus and preeclampsia) or fetal structural and congenital abnormalities were considered as elimination criteria. All participants completed a medical/obstetric history and demographic questionnaire at enrollment. Self-reported pregestational weight and height measured at the first visit were used to calculate the maternal pBMI using the formula weight (kg)/[height (m^2^)]. Women were classified as having normal weight, overweight, or obesity according to the criteria set by the World Health Organization (28). Pregestational weight was estimated through a structured interview, which gave us a more accurate approximation. Maternal body weight was also measured at enrollment. Maternal GWG was determined as the difference between the last recorded weight before delivery and the self-reported pregestational weight and classified as insufficient, adequate and excessive according to the Institute of Medicine criteria (29).

### 2.2 Sample collection

Maternal blood samples were obtained after an 8-hour fast at enrollment and follow-up. Blood samples were centrifuged at 400 g for 15 minutes, and serum was frozen at -70°C until assayed.

### 2.3 Biochemical analysis

Serum glucose, total cholesterol, low-density lipoproteins (LDL), and high-density lipoproteins (HDL), and triglyceride concentrations were measured by enzymatic colorimetric methods utilizing an automated analyzer (ISE Echo Lory 2000) and commercial kits (DiaSys Diagnostic Systems GmbH, Holzheim, Germany). Hemoglobin A1c (HbA1c) measurement was performed by turbidimetric immunoassay using an automated analyzer (InnovaStar) and commercial kits (DiaSys Diagnostic Systems GmbH, Germany). Insulin was measured on an ARCHITECT i1000SR Clinical Chemistry Analyzer (Abbot Diagnostics, Abbott Park, IL, USA). Insulin resistance was determined by applying the homeostasis model assessment formula: HOMA-IR = [fasting insulin (µU/ml) × fasting plasma glucose (mg/dl)]/405. PGRN, AFABP, FGF21, and BDNF maternal concentrations were determined utilizing DuoSet ELISA kits (R&D Systems, Inc., Minneapolis, MN, USA).

### 2.4 Neonate measurements

Weight and length were measured at birth and one month later using a soft tape measure and a baby scale. The neonate was classified as small for gestational age (SGA), adequate for gestational age (AGA), or large for gestational age (LGA) based on Intergrowth-21 parameters (30). Low birth weight was defined as any birth weight <2.5 kg. At one month, the percent fat mass was determined by qualified personnel using air displacement pletismography (PEA POD system, COSMED USA, Inc., Concord, CA, USA).

### 2.5 Statistical analysis

Statistical analysis was performed with the IBM SPSS Statistics 27.0 program (IBM SPSS Inc., Chicago, IL). Data distribution was assessed using the Kolmogorov-Smirnov test. For variables that deviated significantly from normality, rank-based inverse normal transformations were applied. Quantitative variables are presented as mean ± standard deviation. Repeated measures ANOVA was used to compare biochemical and organokine measurements among trimesters of pregnancy. One-way ANOVA with *post-hoc* Tukey was used to compare these same variables between women classified by pBMI and GWG. Pearson’s test was used to calculate bivariate correlations. Stepwise multiple linear regression models were performed to determine which variables were independently associated with neonatal anthropometrics and adiposity. Categorical variables are presented as counts and percentages. The Chi-square test was used for the bivariate analysis of the categorical variables. *p <*0.05 was defined as statistically significant.

## 3 Results

One hundred mother-neonate dyads were studied. **Table 1** shows the clinical characteristics of the participants. As expected, there were significant differences in pBMI according to pregestational nutritional status. In women with normal weight, maternal weight was found to be significantly higher in the first trimester than self-reported weight (**Supplementary Table 1**). As this could introduce bias the GWG classification in this group, GWG classification was performed using self-reported weight. GWG and one-month-old neonate weight were lower in women with obesity than in women with normal weight. In the rest of the studied variables, the groups were homogeneous.

**Table 1:** Clinical characteristics of the study population.

	All women (n=100)	Normal weight (n=40)	Overweight (n=31)	Obesity (n=29)	*p*
Maternal age – years	29.9 ± 5.2	29.5 ± 5.3	29.8 ± 5.3	30.7 ± 4.9	NS
pBMI – kg/m^2^	27.6 ± 5.7	22.7 ± 1.6	27.6 ± 1.4	34.3 ± 4.1	<0.001^a^
Gestational weight gain – kg	7.7 ± 4.5	8.9 ± 4.9	6.1 ± 4.3	4.6 ± 4.1	<0.001^b^
Gestational weight gain classification – n (%)					
Insufficient Adequate Excessive	59.0 (59.0%) 28.0 (28.0%) 13.0 (13.0%)	26.0 (65.0) 9.0 (22.5) 5 (12.5)	18.0 (58.0) 10.0 (32.3) 3.0 (9.7)	15.0 (51.6) 9.0 (31.0) 5.0 (17.3)	NS
Gestational age at birth – weeks	38.9 ± 1.1	39.2 ± 1.0	38.6 ± 1.0	38.9 ± 1.1	NS
Parity– n (%)					
Primiparous Multiparous	69.0 (69.0) 31.0 (31.0)	29.0 (72.5) 11.0 (27.5)	21.0 (67.7) 10.0 (32.3)	19.0 (65.5) 10.0 (34.5)	NS
Delivery mode – n (%)					
Vaginal C-section	50.0 (50.0) 50.0 (50.0)	22.0 (55.0) 18.0 (45.0)	13.0 (41.9) 18.0 (58.1)	15.0 (51.7) 14.0 (48.3)	NS
Neonatal sex – n (%)					
Female Male	41.0 (41.0) 59.0 (59.0)	16.0 (40.0) 24.0 (60.0)	18.0 (58.1) 13.0 (41.9)	14.0 (48.3) 15.0 (51.7)	NS
Birth weight – kg	3.0 ± 0.34	3.0 ± 0.31	2.9 ± 0.36	2.9 ± 0.36	NS
Low birth weight – n (%)	8.0 (8.0)	0 (0)	4.0 (13.0)	4.0 (14.0)	NS
Birth length – cm	47.3 ± 1.6	47.6 ± 1.4	47.0 ± 1.5	47.1 ± 1.91	NS
Birth weight classification – n (%)					
SGA AGA LGA	18.0 (18.0) 81.0 (81.0) 1.0 (1.0)	4.0 (10.0) 35.0 (87.0) 1.0 (3.0)	8.0 (25.8) 23.0 (74.2) 0 (0)	7.0 (24.1) 22.0 (75.9) 0 (0)	NS
One-month-old neonate weight – kg	4.1 ± 0.6	4.3 ± 0.5	4.1 ± 0.6	3.9 ± 0.6	0.018^b^
One-month-old neonate length – cm	51.8 ± 5.4	53.4 ± 5.3	50.5 ± 4.6	51.4 ± 6.1	NS
One-month-old neonate fat mass percentage – %	14.3 ± 4.7	14.3 ± 4.6	14.7 ± 4.8	13.3 ± 5.4	NS

pBMI, pregestational body mass index; SGA, small for gestational age; AGA, adequate for gestational age; LGA, large for gestational age; NS, not significant.

^a^ *p* value for all possible comparisons among the groups.

^b^ *p* value only for comparison between normal weight and obesity. Other comparisons were not significant.

Biochemical changes characteristic of pregnancy were observed in all women, including increased insulin concentrations, heightened insulin resistance, and increased lipid concentrations toward the end of pregnancy (**Table 2**). No significant differences in biochemical variables were observed according to pre-pregnancy nutritional status or GWG classification (**Supplementary Tables 2**, **3**).

**Table 2:** Biochemical characteristics of the study population.

	First trimester (n=100)	Second trimester (n=100)	Third trimester (n=100)	*p*
Fasting glucose – mg/dL	80.2 ± 12.5	77.6 ± 12.8	77.9 ± 11.7	NS
Fasting insulin – μU/mL	9.0 ± 4.3	Data not available	14.5 ± 6.0	0.006^a^
HOMA-IR	1.9 ± 1.1	Data not available	3.1 ± 1.6	0.042^a^
Triglycerides – mg/dL	134.9 ± 47.2	181.9 ± 91.1	217.6 ± 65.1	<0.001^b^
Total cholesterol – mg/dL	176.2 ± 34.8	208.0 ± 44.2	235.9 ± 45.7	<0.001^b^
HDL cholesterol – mg/dL	55.8 ± 12.3	60.1 ± 12.6	61.6 ± 14.5	0.002^c^
LDL cholesterol – mg/dL	86.6 ± 22.7	106.1 ± 26.2	121.3 ± 36.2	<0.001^b^
HbA1c – %	5.1 ± 0.6	4.9 ± 0.7	5.0 ± 0.9	<0.001^d^

HOMA-IR, homeostasis model assessment – insulin resistance; HDL, high-density lipoprotein; LDL, low-density lipoprotein; HbA1c, hemoglobin A1c; NS, not significant.

^a^ *p* value for comparison between first and third trimesters. The other comparisons were not significant.

^b^ *p* value for all possible comparisons among first,second,and third trimesters.

^c^ *p* value for comparison between first and second trimesters,and first and third trimesters. The other comparisons were not significant.

^d^ *p* value for comparison between first and second trimesters. The other comparisons were not significant.

**Figure 1:**
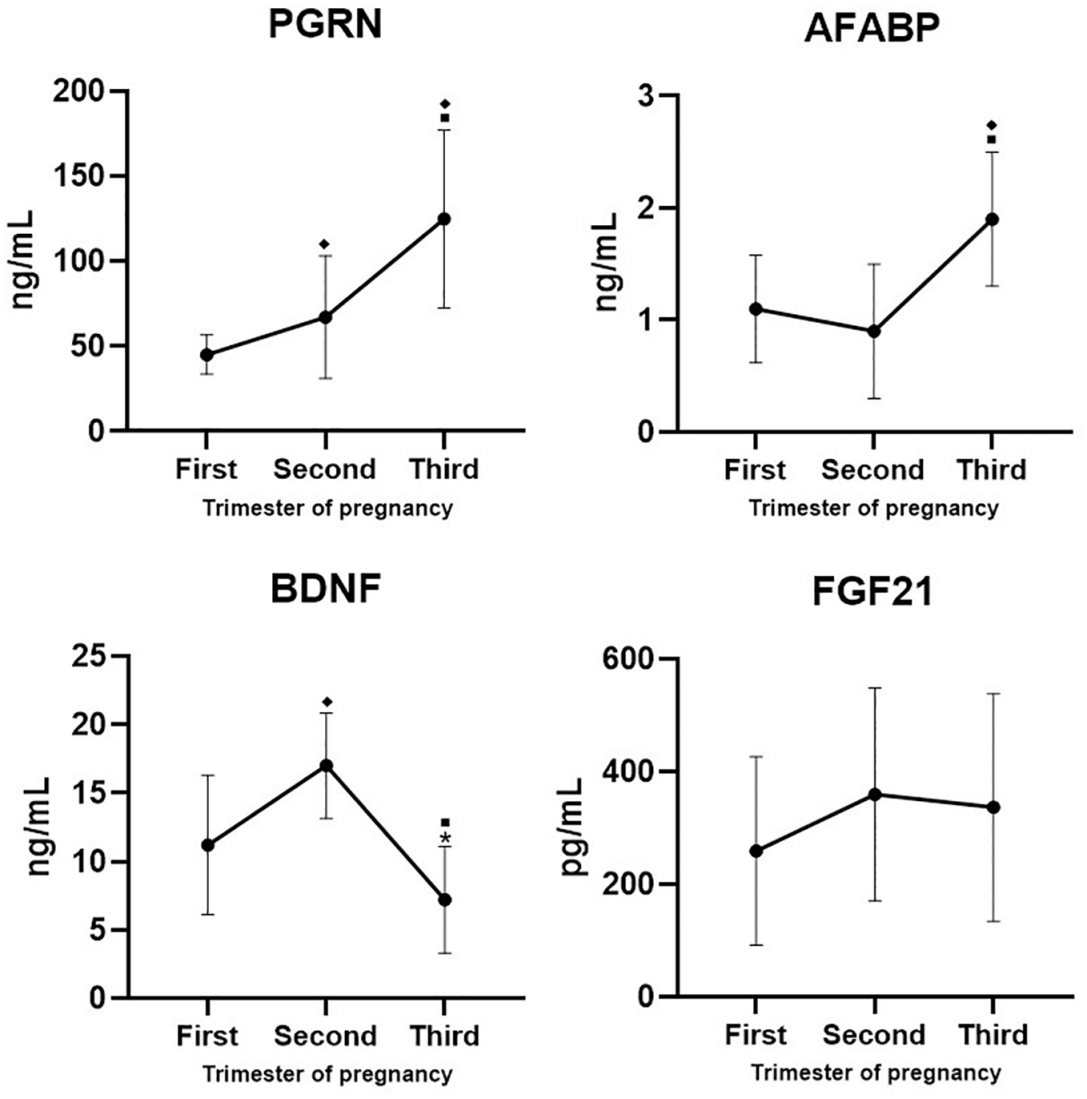
Organokine concentrations throughout pregnancy in all participants. ♦*p*<0.001 vs first trimester, ▪*p*<0.001 vs second trimester, *p=0.003 vs first trimester of pregnancy. PGRN, progranulin; AFABP, adipocyte-specific fatty acid-binding protein; FGF21, fibroblast growth factor 21; BDNF, brain-derived neurotrophic factor. *n*=100.

Regarding organokines, PGRN concentrations significantly increased as pregnancy progressed, while AFABP concentrations increased until the third trimester and the highest BDNF concentrations were observed in the second trimester. In contrast, FGF21 concentrations did not change during pregnancy (**Figure 1**).

According to pregestational nutritional status, first- and second-trimester AFABP concentrations were higher in women with obesity than in normal-weight and overweight women, and third-trimester FGF21 concentrations were lower in women with obesity than in normal-weight women (**Supplementary Table 4**). No significant differences were observed in organokine concentrations based on GWG classification (**Supplementary Table 5**).

In all women, birth weight positively correlated with GWG (r=0.237, *p*=0.024) and negatively with third-trimester AFABP concentration (r=-0.265, *p*=0.024). Birth length positively correlated with third-trimester PGRN concentration (r=0.229, *p*=0.029). One-month-old neonate weight positively correlated with second- and third-trimester PGRN concentrations (r=0.274, *p*=0.021, and r=0.252, *p*=0.03, respectively), and negatively with pBMI (r=-0.354, *p*=0.002). The percentage of one-month-old neonate fat mass negatively correlated with pBMI (r=-0.268, *p*=0.021).

Models with maternal clinical variables and organokine concentrations that significantly predicted neonatal characteristics are shown in **Table 3**. Third-trimester PGRN concentration contributed to the prediction of weight and length at birth, while first- and third-trimester BDNF concentrations contributed to the prediction of birth length and one-month old neonate length, respectively. Second-trimester FGF21 concentrations and third-trimester AFABP concentrations contributed to the prediction of one-month-old neonate fat mass percentage.

**Table 3:** Multiple regression analysis for neonatal outcomes.

Outcome	Model	Beta	Standardized beta coefficient	95% CI	*p*	R	Adjusted R^2^	*p*
Birth weight	Gestational age at birth Maternal age Third-trimester PGRN concentration	0.11 -0.033 0.003	0.38 -0.61 0.53	(0.026, 0.186) (-0.05, -0.017) (0.001, 0.005)	0.013 <0.001 0.002	0.82	0.62	<0.001
Birth length	Gestational age at birth Maternal age Third-trimester PGRN concentration First-trimester BDNF concentration	0.76 -0.21 0.024 0.06	0.43 -0.60 0.62 0.28	(0.31, 1.2) (-0.30, -0.12) (0.014, 0.035) (0.004, 0.12)	0.002 <0.001 <0.001 0.037	0.88	0.72	<0.001
One-month-old neonate length	Maternal age Third-trimester BDNF concentration	-1.03 0.45	-0.71 0.40	(-1.5, -0.57) (0.1, 0.81)	<0.001 0.017	0.82	0.63	<0.001
One-month-old neonate fat mass percentage	pBMI GWG Second-trimester FGF21 concentration Third-trimester AFABP concentration	-0.58 -0.32 -0.004 -1.27	-0.86 -0.39 -0.39 -0.42	(-0.8, -0.4) (-0.6, -0.05) (-0.008, -0.001) (-2.1, -0.4)	<0.001 0.025 0.012 0.007	0.82	0.59	<0.001

PGRN, progranulin; AFABP, adipocyte-specific fatty acid-binding protein; FGF21, fibroblast growth factor 21; BDNF, brain-derived neurotrophic factor; pBMI, pregestational body mass index; GWG, gestational weight gain. The models included the following variables, maternal age, pBMI, GWG, gestational age at birth, and organokine concentrations throughout pregnancy. Only the significant variables are shown in each model. None of the variables was significant for the prediction of neonatal length at one month of life. *n*=100 mother-neonate dyads.

## 4 Discussion

This is the first study to describe changes in maternal PGRN and AFABP concentrations throughout pregnancy. Furthermore, this is the first study to document that, in conjunction with maternal variables, maternal concentrations of PGRN, AFABP, BDNF, and FGF21 significantly predict neonatal weight, length, and fat mass percentage. Interestingly, maternal obesity and excessive gestational weight gain had no significant effect on the concentrations of these organokines, with the exception of AFABP in the first and second trimesters, which presented higher levels in women with pregestational obesity. This can be attributed to the observation that these groups exhibited moderate metabolic alterations, as evidenced by the biochemical results and the absence of severe maternal and neonatal complications.

We found that maternal PGRN concentrations increase as pregnancy progresses and that its third-trimester concentrations positively correlated with birth length, while its second- and third-trimester concentrations positively correlated with one-month-old neonate weight. Moreover, maternal PGRN concentrations in the third trimester contribute positively to the prediction of neonatal length and weight at birth. In a murine model, PGRN administration alters glucose tolerance and insulin sensitivity, favoring insulin resistance (31). As mentioned, individuals with type 2 diabetes or obesity have increased plasma concentrations of PGRN (17). It is plausible that the progressive elevation in maternal PGRN levels documented in our investigation may be linked to the physiological insulin resistance characteristic of pregnancy, and thus influence the availability of glucose for fetal growth; however, no differences in maternal concentrations of this organokine have been observed between women with gestational diabetes mellitus and controls (32, 33). Further research is needed to elucidate these aspects.

AFABP is a protein mainly secreted by adipose tissue (34). This is consistent with our finding that women with pregestational obesity had higher concentrations of this organokine than pregestational normal weight and overweight women in the first and second trimesters of gestation; however, these differences disappear in the third trimester. The latter can be explained by the fact that women with pregestational obesity start pregnancy with more visceral adipose tissue, but increases little throughout pregnancy, while women with normal weight gain more visceral adipose tissue through pregnancy. The difference in visceral adipose tissue between these two groups is minimal in the third trimester of pregnancy (35). We observed a negative correlation between third-trimester AFABP concentration and birth weight. These concentrations also contributed negatively to the prediction of the percentage of fat mass in the newborn. The correlation has already been reported in another study (36); however, further studies are needed to elucidate the mechanisms that may be involved in the connection between this maternal organokine and neonatal adiposity. As a perspective, it is important to study vaspin levels throughout pregnancy, as it has been suggested that it regulates glucose and lipid metabolism, and circulating levels of AFABP have been observed to positively influence its maternal concentrations (37).

In pregnancy, BDNF has been mainly studied in the context of perinatal depression (38). It has been reported that its maternal concentrations decrease as pregnancy progresses (39). In contrast, we observed that maternal BDNF concentrations are higher in the second trimester than in the first and third trimesters, and are significantly lower in the third than in the first trimester. Further studies are needed to elucidate changes in maternal concentrations of this organokine, taking into account that circulating BDNF levels are influenced by a non-fasting state, age, residence in an urban area, mental state and ethnicity (39, 40). Furthermore, first- and third-trimester BDNF concentrations positively contribute to the prediction of birth length and neonatal adiposity, respectively. Currently, our understanding of the underlying mechanisms of these observations is limited.

It has been reported that in healthy pregnant women FGF21 concentrations are significantly higher in the third trimester than in the first and second trimesters. In our study, we observed that the concentrations of this organokine did not differ throughout pregnancy (41). This inconsistency may be due to differences in the study population, sample size (100 *vs* 52 participants in the previous study), or the marked difference in the range established for third-trimester gestational age (28-34 vs. 33.4-38.6 weeks). We observed that second-trimester FGF21 negatively contribute to the prediction of neonatal fat mass percentage. Nevertheless, we currently lack the necessary information to explain the impact of this maternal organokine on neonatal adiposity.

Although our findings are consistent with other studies on the correlations of maternal organokines and neonatal birth weight, they have limitations. First, its design does not allow for the establishment of causal relationships among maternal organokine concentrations with neonatal anthropometry and adiposity. Also, for the secondary analyses according to pBMI and GWG, the sample size was small for some groups. Finally, our findings should be interpreted cautiously as they must be validated in a wider range of birth weight that includes a larger number of SGA and LGA neonates. The strength of our study design is that it is a longitudinal study of women with complete clinical follow-up and without any metabolic disease beyond excessive maternal weight, as evidenced by the biochemical results.

## 5 Conclusion

Maternal PGRN, AFABP, and BDNF concentrations, but not FGF21, vary throughout pregnancy. These organokines and maternal characteristics can be useful in the prediction of neonatal weight, length, and percentage fat mass. Our findings open a new field of research on the role of maternal organokines in fetal programming.

## Data Availability

The original contributions presented in the study are included in the article/**Supplementary Material**. Further inquiries can be directed to the corresponding author.
