# Supplementary material for: Maternal organokines throughout pregnancy as predictors of neonatal anthropometric characteristics and adiposity
Source: Front Endocrinol (Lausanne). 2024 Dec 4;15:1423950. doi: 10.3389/fendo.2024.1423950 (PMC11653021; doi:10.3389/fendo.2024.1423950)
Supplement: Supplementary file 1 [file Table1.docx]

Supplementary Material

# Supplementary Tables

Supplementary Table 1. Comparison between self-reported weight and weight measured in the first trimester.

|  | Pregestational weight – kg | Weight at 1st visit – kg | *p* |
| --- | --- | --- | --- |
| All women n=100 | 67.8±14.5 | 68.2±13.5 | 0.21 |
| Normal weight n=40 | 56.8±5.6 | 58.8±6.7 | 0.01 |
| Overweight n=31 | 67.1±4.5 | 67.4±4.6 | 0.49 |
| Obesity n=29 | 83.6±15.5 | 82.8±14.2 | 0.20 |

Supplementary Table 2. Biochemical characteristics by pregestational nutritional status.

|  | Normal weight  n=40 | Overweight  n=31 | Obesity  n=29 |
| --- | --- | --- | --- |
| Fasting glucose – mg/dL  1^st^ T  2^nd^ T  3^rd^ T | 78.0±13.8  75.2±12.0  77.8±12.7 | 80.9±9.9  77.7±11.6  75.0±10.3 | 82.5±12.8  81.1±14.5  81.2±11.3 |
| Fasting insulin – μU/mL  1^st^ T  2^nd^ T  3^rd^ T | 7.3±4.2  Data not available  15.8±7.9 | 9.1±4.4  Data not available  13.3±5.7 | 11.3±4.4  Data not available  13.9±5.4 |
| HOMA-IR  1^st^ T  2^nd^ T  3^rd^ T | 1.5±0.7  Data not available  3.4±1.9 | 1.9±1.0  Data not available  2.7±1.6 | 2.5±1.5  Data not available  2.9±0.9 |
| Triglycerides – mg/dL  1^st^ T  2^nd^ T  3^rd^ T | 136.1±42.2  182.8±94.4  220.5±66.8 | 130.5±54.2  193.6±94.0  216.2±69.5 | 138.1±47.2  168.6±84.7  215.0±59.9 |
| HDL – mg/dL  1^st^ T  2^nd^ T  3^rd^ T | 53.9±11.7  59.4±11.3  61.0±13.1 | 60.1±13.5  62.6±14.7  63.9±15.1 | 54.0±11.2  58.4±11.9  60.1±16.0 |
| LDL – mg/dL  1^st^ T  2^nd^ T  3^rd^ T | 84.5±23.6  106.2±25.3  117.5±31.0 | 92.6±21.9  111.5±27.0  131.6±49.4 | 83.5±21.6  100.3±26.3  115.8±22.7 |
| Total cholesterol – mg/dL  1^st^ T  2^nd^ T  3^rd^ T | 170.1±31.6  207.3±42.1  236.550.1 | 181.9±38.2  219.0±52.5  238.0±43.8 | 178.7±35.1  197.2±35.8  232.9±42.5 |
| HbA1c – %  1^st^ T  2^nd^ T  3^rd^ T | 5.1±0.6  4.9±0.8  5.1±1.1 | 5.1±0.6  4.7±0.5  4.7±0.7 | 5.1±0.5  5.0±0.7  5.1±0.7 |

^HOMA-IR: homeostasis model assessment – insulin resistance; HDL: high-density lipoprotein; LDL: low-density lipoprotein; HbA1c: hemoglobin A1c; T: trimester. No significant differences were observed among the groups.^

Supplementary Table 3. Biochemical characteristics by gestational weight gain classification.

|  | Insufficient GWG  n=59 | Adequate GWG  n=28 | Excessive GWG  n=13 |
| --- | --- | --- | --- |
| Fasting glucose – mg/dL  1^st^ T  2^nd^ T  3^rd^ T | 79.5±12.1  76.7±13.4  78.0±12.7 | 82.5±13.4  81.7±13.5  79.5±12.2 | 79.4±10.9  74.4±11.2  75.2±8.8 |
| Fasting insulin – μU/mL  1^st^ T  2^nd^ T  3^rd^ T | 8.4±4.6  Data not available  13.6±6.4 | 7.1±3.9  Data not available  17.7±7.0 | 14.1±5.2  Data not available  12.9±4.5 |
| HOMA-IR  1^st^ T  2^nd^ T  3^rd^ T | 1.6±0.7  Data not available  3.0±1.8 | 2.1±1.6  Data not available  3.7±1.8 | 2.8±1.5  Data not available  2.5±1.0 |
| Triglycerides – mg/dL  1^st^ T  2^nd^ T  3^rd^ T | 135.8±49.8  189.9±98.2  217.2±67.3 | 133.2±46.7  175.2±87.5  212.8±72.6 | 136.0±39.5  176.2±70.9  215.4±41.9 |
| HDL – mg/dL  1^st^ T  2^nd^ T  3^rd^ T | 54.8±13.2  58.8±13.2  62.4±12.5 | 58.9±11.9  63.7±12.2  60.4±15.9 | 57.6±11.5  61.9±12.0  66.6±17.0 |
| LDL – mg/dL  1^st^ T  2^nd^ T  3^rd^ T | 86.5±23.6  104.4±26.3  127.1±42.3 | 90.4±25.2  115.0±27.8  114.7±28.9 | 84.3±12.8  103.0±24.7  115.2±27.2 |
| Total cholesterol – mg/dL  1^st^ T  2^nd^ T  3^rd^ T | 176.8±32.2  205.0±45.3  237.2±47.0 | 184.5±35.6  219.4±49.6  231.9±42.3 | 169.8±40.9  213.5±32.3  245.8±52.3 |
| HbA1c – %  1^st^ T  2^nd^ T  3^rd^ T | 5.0±0.6  4.8±0.6  4.9±1.0 | 5.2±0.6  5.0±0.7  5.1±0.8 | 5.2±0.6  5.0±0.9  5.1±0.7 |

^GWG: gestational weight gain; HOMA-IR: homeostasis model assessment – insulin resistance; HDL: high-density lipoprotein; LDL: low-density lipoprotein; HbA1c: hemoglobin A1c; T: trimester. No significant differences were observed among the groups.^

Supplementary Table 4. Organokine maternal concentrations through pregnancy by pregestational nutritional status.

|  | Normal weight  n=40 | Overweight  n=31 | Obesity  n=29 | *p* |
| --- | --- | --- | --- | --- |
| PGRN  1^st^ T  2^nd^ T  3^rd^ T | 46.9±10.0  72.6±29.2  137.1±49.5 | 46.6±11.3  61.3±18.0  125.0±60.8 | 40.9±13.1  64.9±54.5  107.2±42.9 | NS  NS  NS |
| AFABP  1^st^ T  2^nd^ T  3^rd^ T | 0.9±0.5*  0.7±0.5*  1.9±1.2 | 0.9±0.6*  0.6±0.4*  1.8±1.1 | 1.6±0.7*  1.4±0.9*  2.0±1.2 | 0.002 (NW vs O and OW vs O)  0.012 (NW vs O); 0.015 (OW vs O)  NS |
| FGF21  1^st^ T  2^nd^ T  3^rd^ T | 329.3±175.8  438.3±205.0  587.3±200.8* | 115.3±89.3  390.5±221.3  257.1±152.1 | 319.6±107.8  233.3±129.0  126.0±98.2* | NS  NS  0.014 (NW vs O) |
| BDNF  1^st^ T  2^nd^ T  3^rd^ T | 10.0±5.8  17.9±4.5  6.4±4.0 | 11.8±6.4  16.1±4.5  8.4±5.4 | 12.1±5.5  16.5±4.4  7.0±3.9 | NS  NS  NS |

^PGRN: progranulin; AFABP: adipocyte-specific fatty acid-binding protein; FGF21: fibroblast growth factor 21; BDNF: brain-derived neurotrophic factor; T: trimester; NS: not significant; NW: normal weight; OW: overweight; O: obesity.^

Supplementary Table 5. Organokine maternal concentrations through pregnancy by gestational weight gain classification.

|  | Insufficient GWG  n=59 | Adequate GWG  n=28 | Excessive GWG  n=13 |
| --- | --- | --- | --- |
| PGRN  1^st^ T  2^nd^ T  3^rd^ T | 47.2±11.2  64.4±21.2  133.7±57.2 | 43.6±11.9  73.7±54.9  118.0±51.4 | 44.0±9.3  67.1±30.1  114.9±37.2 |
| AFABP  1^st^ T  2^nd^ T  3^rd^ T | 1.0±0.4  0.7±0.5  2.1±1.0 | 1.2±0.5  0.8±0.7  1.5±0.9 | 0.8±0.5  1.1±0.5  1.3±0.8 |
| FGF-21  1^st^ T  2^nd^ T  3^rd^ T | 234.6±166.8  253.3±184.7  398.0±166.0 | 221.0±152.4  324.3±173.1  227.6±156.0 | 342.0±224.8  371.0±200.2  335.0±194.8 |
| BDNF  1^st^ T  2^nd^ T  3^rd^ T | 11.3±6.0  17.3±4.6  7.4±4.3 | 11.1±5.7  15.9±4.7  6.8±5.1 | 9.3±5.5  18.9±6.3  6.9±4.1 |

^GWG: gestational weight gain; PGRN: progranulin; AFABP: adipocyte-specific fatty acid-binding protein; FGF21: fibroblast growth factor 21; BDNF: brain-derived neurotrophic factor; T: trimester. No significant differences were observed between the groups.^
